# Supplementary material for: RNA-Seq Provides Insights into the Mechanisms Underlying Ilyonectria robusta Responding to Secondary Metabolites of Bacillus methylotrophicus NJ13
Source: J Fungi (Basel). 2022 Jul 26;8(8):779. doi: 10.3390/jof8080779 (PMC9332032; doi:10.3390/jof8080779)
Supplement: Supplementary file 1 [file jof-08-00779-s001.zip › jof-1810617-supplementary.pdf]

Journal of *Fungi*

MDPI

Supplementary Materials

Table S1. Primers used in qRT-PCR.

Gene id		Primer		
evm.TU.Contig4.662	F*	5'	GCTTCACCGACCGCTACAACTG	3'
	R*	5'	CAGCAGCAAGCAGGTTCGAGATAC	3'
evm.TU.Contig2.1111	F	5'	ACGAGAGCAGAGTGTCCAAGGAG	3'
	R	5'	GTTGACATCGGCGGAGAGGTTG	3'
evm.TU.Contig2.1123	F	5'	GGGAGAGACGGGAGCCTACATTC	3'
	R	5'	ACATCATCCTCACTCGGTCCAGAG	3'
evm.TU.Contig5.810	F	5'	GATCGCTCCAACTCGCCTCAAC	3'
	R	5'	CGGCAGTTCGCTTCAAGACCTC	3'
evm.TU.Contig30.108	F	5'	CATCATCAGCGACTTCACCGTCTC	3'
	R	5'	GGATGTCACCGTTGGAAGCAGAG	3'
evm.TU.Contig17.77	F	5'	TGGTATGGATGGTCGGTGGAGAG	3'
	R	5'	AGAGGCGGCGTAGGTGATGTAG	3'
evm.TU.Contig9.224	F	5'	CACTGCTTTGTTTGCGGGAATCAC	3'
	R	5'	CGATGTGGAACGAGTTGGTGTAGG	3'
evm.TU.Contig20.314	F	5'	GCCCTGGTTTCATCTGCCTACAC	3'
	R	5'	ACCGAGAACAAACAGCGAGACAC	3'
evm.TU.Contig1.616	F	5'	CCAACGGCACTGACATCCACTG	3'
	R	5'	AGAGAAAGAAACACCAGGACAAGGC	3'
evm.TU.Contig16.341	F	5'	ATATACGCCTCCCTCCGAGTTAGTG	3'
	R	5'	AGCCGACAATATGATGCCTGTTACC	3'
evm.TU.Contig19.352	F	5'	TCACGAACTGGAGGGCTTAGAGG	3'
	R	5'	CCGTCATCTGCCACTTCTACATTCC	3'
evm.TU.Contig23.290	F	5'	CTTGCTGAGGTCAACGGCTATGG	3'
	R	5'	ACGAGGAACAGAGGGAACTGGAAG	3'
evm.TU.Contig18.225	F	5'	ACTCGGATGTTTGGGTTGGTTGG	3'
	R	5'	GTCATACGCAACGCCAGTAGAGG	3'
evm.TU.Contig13.313	F	5'	CGCTGCTCTTATCGCTGCTCTC	3'
	R	5'	ACTTCTTCATGCCACCATCGTAGC	3'
novel.1805	F	5'	TCTTGGCTTGGCTGGATCTTGAATG	3'
	R	5'	TGTGGTTGGATAAAACGACGGGAAG	3'
novel.2288	F	5'	CGGCTATGTTGTTGCGGAGGAC	3'
	R	5'	CAACCTTACCGGAACCGAGAAGTG	3'
novel.2293	F	5'	CCGTTCTTCTTGCCGTCACTCC	3'
	R	5'	TTCAAGGATGCGAAGCCGTCAG	3'

* Forward primer or Reversed primer

Table S2. Quality analysis of RNA-Seq sequencing data.

Sample	Raw reads	Clean reads	Clean ba-ses	Error rate(%)	Q20 (%)	Q30 (%)	GC (%)	Total map
BF_R4d_1	49316358	48151054	7.22G	0.02	98.16	94.6	56.3	47254765(98.14%)
BF_R4d_2	48971862	47305862	7.1G	0.02	98.11	94.63	55.9	46257232(97.78%)
BF_R4d_3	49669084	48647556	7.3G	0.02	98.12	94.52	55.89	47745077(98.14%)
BF_R7d_1	47309784	46204562	6.93G	0.02	98.2	94.68	56.49	45361811(98.18%)
BF_R7d_2	48575250	47496712	7.12G	0.02	98.15	94.59	56.54	46717545(98.36%)
BF_R7d_3	48963314	47962720	7.19G	0.03	97.8	93.73	56.64	47058908(98.12%)
R4d_1	48905388	47588660	7.14G	0.02	98.09	94.49	55.57	46621126(97.97%)
R4d_2	47784000	46263794	6.94G	0.02	98.08	94.42	55.18	45376497(98.08%)
R4d_3	47735346	46341570	6.95G	0.02	98.19	94.73	55.51	45502131(98.19%)
R7d_1	50522322	49517844	7.43G	0.02	98.12	94.49	56.07	48434677(97.81%)
R7d_2	55207722	54225066	8.13G	0.02	98.25	94.78	56.52	53480130(98.63%)
R7d_3	47240898	46161110	6.92G	0.02	98.21	94.71	56.51	45376885(98.3%)

Table S3. Gene annotation and expression analysis of potential response genes in strain CBLJ-3.

Gene id	Log2 (Fold change)		Padj		Gene description
	BF_R4d vs R4d	BF_R7d vs R7d	BF_R4d vs R4d	BF_R7d vs R7d	
**Genes encoding glucose transporter**					
evm.TU.Contig22.20	−1.227306797	-	0.006217354	-	Hexose transporter 13 / Multicopy suppressor of snf4 deficiency protein 1 / Sugar (and other) transporter
evm.TU.Contig18.121	−1.091109424	-	0.009672643	-	High-affinity glucose transporter RGT2 / Sugar (and other) transporter
evm.TU.Contig13.217	−1.115730235	-	0.010275477	-	High-affinity glucose transporter SNF3 / Sugar (and other) transporter
evm.TU.Contig7.598	−1.54689462	-	0.011735671	-	High-affinity glucose transporter HXT2 / Sugar (and other) transporter
evm.TU.Contig5.632	−1.449666379	-	0.048741531	-	Probable glucose transporter rco-3 / Sugar (and other) transporter
evm.TU.Contig2.918	-	-1.725870496	-	3.13519E−06	Hexose transporter 1 / Sugar (and other) transporter
**Related genes to damage fungal cell wall**					
evm.TU.Contig11.337	1.945310073	-	3.71275E−13	-	Probable endo-β-1,4-glucanase D / Glycosyl hydrolase family 61
evm.TU.Contig16.235	1.813632607	-	1.46054E−09	-	Endo-1,4-β-glucanase IV / Glycosyl hydrolase family 61
evm.TU.Contig28.214	4.830898872	-	3.67092E−09	-	Probable xyloglucan-specific endo-β-1,4-glucanase A / Glycosyl hydrolase family 12
evm.TU.Contig19.313	2.234198949	-	3.24014E−06	-	Probable xyloglucan-specific endo-β-1,4-glucanase A / Glycosyl hydrolase family 12
evm.TU.Contig28.103	3.277833959	-	1.42353E−05	-	Probable xyloglucan-specific endo-β-1,4-glucanase A / Glycosyl hydrolase family 12
evm.TU.Contig18.203	2.025644872	-	0.000281974	-	Glucan endo-1,6-β-glucosidase B / Glycosyl hydrolase family 5
evm.TU.Contig18.225	5.385803234	-	0.000219848	-	Endo-β-1,4-glucanase B / Glycosyl hydrolase family 5
evm.TU.Contig1.720	1.490552348	-	4.73746E−05	-	Probable endo-1,3(4)-β-glucanase / Glycosyl hydrolases family 16
evm.TU.Contig20.16	-	2.925360733	-	5.55525E−05	Chitinase-1 / Glycosyl hydrolases family 18
evm.TU.Contig30.145	-	1.127720541	-	0.039018451	Chitinase-1 / Glycosyl hydrolases family 18

evm.TU.Contig32.86	1.052093729	-	-	0.029231604	Glycosyl hydrolases family 18
evm.TU.Contig19.330	–2.284435178	-	-	1.20E–12	Chitin synthase
evm.TU.Contig1.1596	–2.073600898	-	-	0.010060275	Chitin synthase D / Chitin synthase
**Sterol synthesis / transfer genes**					
evm.TU.Contig23.290	1.420367125	-	3.90E–06	-	Probable Delta(7)-sterol 5(6)-desaturase / C-5 sterol desaturase / Sterol-C5-desaturase
evm.TU.Contig2.306	1.383905973	-	2.28E–05	-	Methylsterol monooxygenase / C-4 methylsterol oxidase
evm.TU.Contig6.165	2.730195482	1.290754477	0.000187141	0.023512876	Sterol uptake control protein 2
evm.TU.Contig6.163	2.64514561	1.579654627	0.000454146	0.0425209	Sterol uptake control protein 2
evm.TU.Contig2.889	4.01990853	–2.140157526	2.73E–24	0.000129215	Sterol 24-C-methyltransferase erg-4 / Delta(24)-sterol C-methyltransferase
evm.TU.Contig17.12	1.033408709	-	0.016699708	-	Sterol 24-C-methyltransferase erg-4 / Delta(24)-sterol C-methyltransferase
evm.TU.Contig97.3	–1.797550935	-	7.58E–21	-	Sterol 3-β-glucosyltransferase UGT80B1 / UDP-glucose: sterol glucosyltransferase 80B1
evm.TU.Contig19.352	–2.038076735	-	1.18E–08	-	Sterol 3-β-glucosyltransferase UGT80A2 / UDP-glucose: sterol glucosyltransferase 80A2
evm.TU.Contig16.341	–1.501800564	-	4.65E–07	-	Sterol 3-β-glucosyltransferase UGT80A2 / UDP-glucose: sterol glucosyltransferase 80A2
evm.TU.Contig29.33	–1.090541494	-	0.001046147	-	Sterol 3-β-glucosyltransferase UGT80A2 / UDP-glucose: sterol glucosyltransferase 80A2
evm.TU.Contig13.242	–1.106797469	-	0.001354601	-	Sterol 3-β-glucosyltransferase UGT80A2 / UDP-glucose: sterol glucosyltransferase 80A2
**Fungal metabolite synthesis genes**					
evm.TU.Contig2.1111	–6.044304189	–3.349124586	1.14E–42	0.008143923	Highly reducing polyketide synthase azaB / HR-PKS azaB
evm.TU.Contig25.194	–1.762052148	-	3.77E–07	-	Reducing polyketide synthase PKS2 / T-toxin biosynthesis protein PKS2

evm.TU.Contig4.114	–1.038261919	-	0.016361089	-	Fusarin C cluster PKS/NRPS FUS1 / Fusarin biosynthesis megasynthetase / Fusarin biosynthesis protein 1
evm.TU.Contig2.1106	–4.873128	–3.191589465	1.03E–18	0.000369083	Nonribosomal peptide synthetase 8
evm.TU.Contig2.1120	–4.881269357	–3.139340497	4.55E–43	0.007720758	Nonribosomal peptide synthetase dtxS1 / Destruxin synthase dtxS1 / Destruxin synthesis protein 1
evm.TU.Contig2.1105	–2.668034775	-	0.000104188	-	Nonribosomal peptide synthetase 12
**Drug resistance genes**					
evm.TU.Contig1.616	1.98265593	-	2.80E–13	-	Major facilitator superfamily
evm.TU.Contig10.57	1.871783393	-	1.26E–08	-	Major facilitator superfamily
evm.TU.Contig20.314	1.517785056	-	3.72E–08	-	Major facilitator superfamily
evm.TU.Contig17.77	2.679730677	-	0.029912132	-	Major facilitator superfamily
evm.TU.Contig24.56	1.932600128	-	0.002120793	-	Pleiotropic ABC efflux transporter of multiple drugs CDR1/CDR ABC transporter
evm.TU.Contig17.72	2.345504487	-	0.000415221	-	Multidrug resistance protein CDR1 / CDR ABC transporter
evm.TU.Contig19.290	3.363687782	-	1.71E–09	-	ABC transporter CDR4 / CDR ABC transporter
evm.TU.Contig5.810	5.280572337	-	0.021739634	-	Multidrug resistance protein CDR2 / CDR ABC transporter
**DEGs of hub genes**					
evm.TU.Contig2.1115	–4.83003	-	7.59E–19	-	L-aspartate decarboxylase dtxS4 / Destruxin synthesis protein 4
evm.TU.Contig23.121	–1.05853	-	0.000384	-	Glutamate/Leucine/Phenylalanine/Valine dehydrogenase
evm.TU.Contig13.145	-	–1.94912	-	3.57E–09	Heat shock protein 90 homolog / Hsp90 protein
evm.TU.Contig3.801	-	–1.77591	-	5.79E–08	Heat shock 70 kDa protein / Hsp70 protein

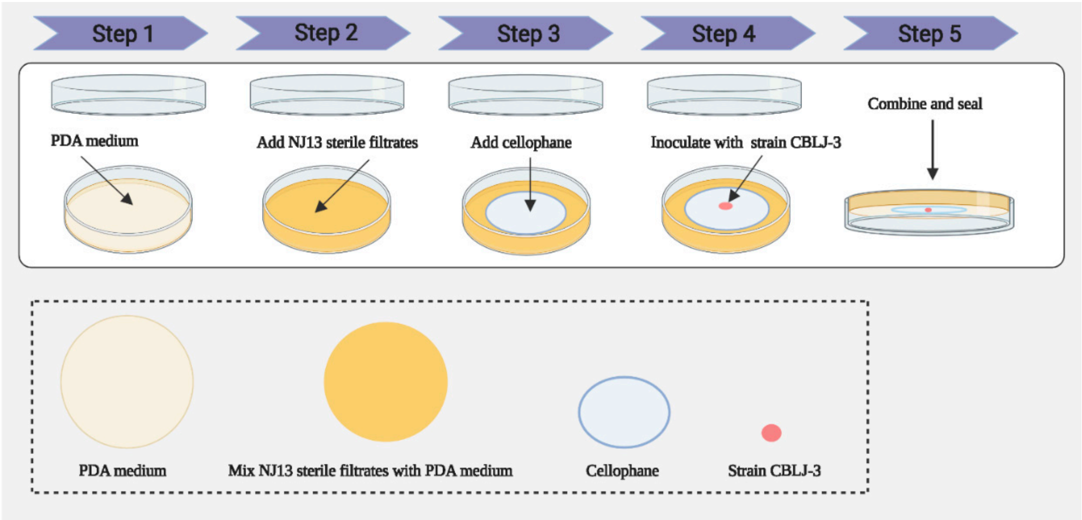

Figure S1. Procedure used for the interaction of CBLJ-3 with NJ13 sterile filtrates. Created with Biorender.com (https://biorender.com, accessed on 9 April 2020).

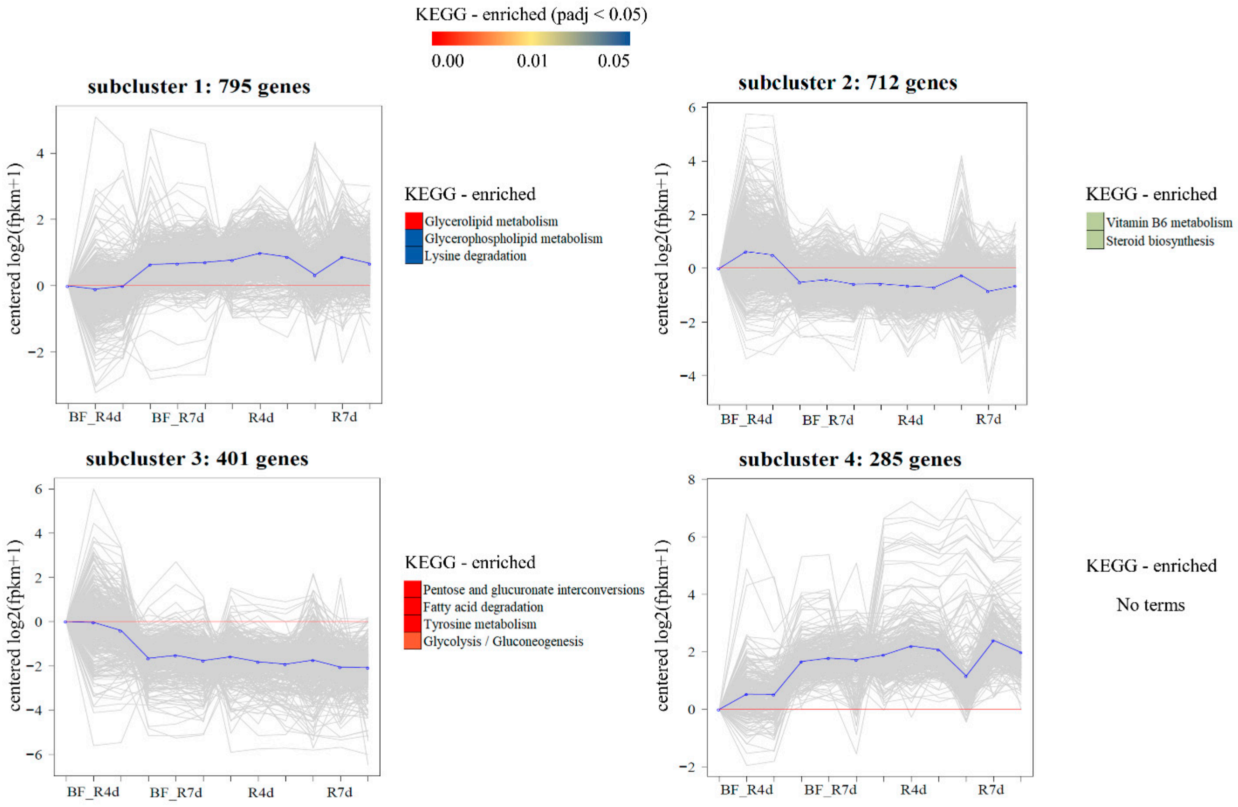

Figure S2. Analysis of KEGG category distribution of DEGs in the four co-expression subclusters constituting the transcriptome of CBLJ-3 under NJ13 sterile filtrates stress. The abscissa is the sample name, and the ordinate is the expression value corrected by the centralized logarithm. The gray line indicates the expression level of genes in the subclusters under different experimental conditions, and the blue line indicates the average expression value of all DEGs in the subcluster under different experimental conditions. By using clusterProfiler R package (Southern Medical University, Guangzhou, China) and p adj < 0.05, the KEGG enrichment analysis was computed.

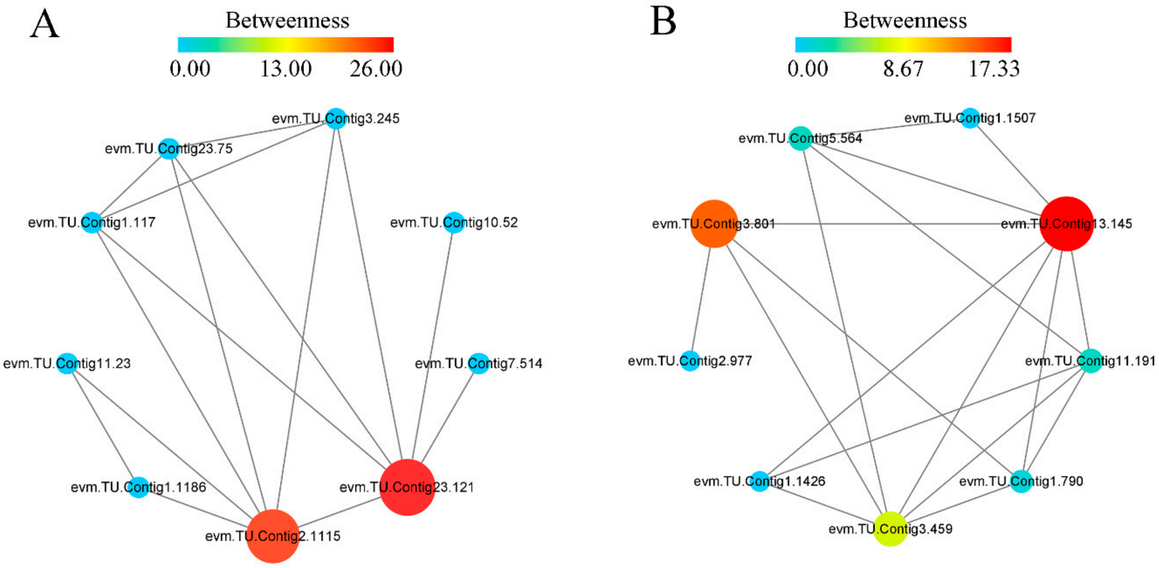

Figure S3. PPI analysis of DEGs at 4 dpi and 7 dpi by the "Betweenness Centrality" method. (A) BF_R4d vs R4d. (B) BF_R7d vs R7d.

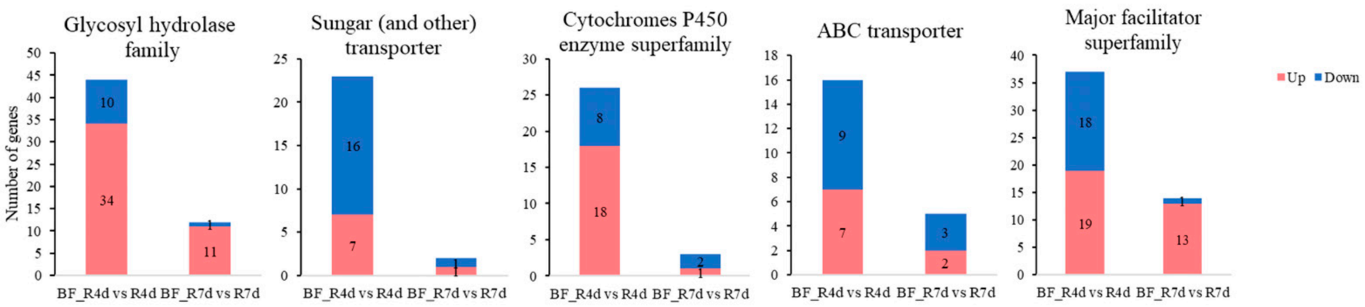

Figure S4. Based on transcriptomic annotation analysis, taxonomic analysis of DEGs. (| $\log_2$ (fold change) | > 1, *p* adj < 0.05).

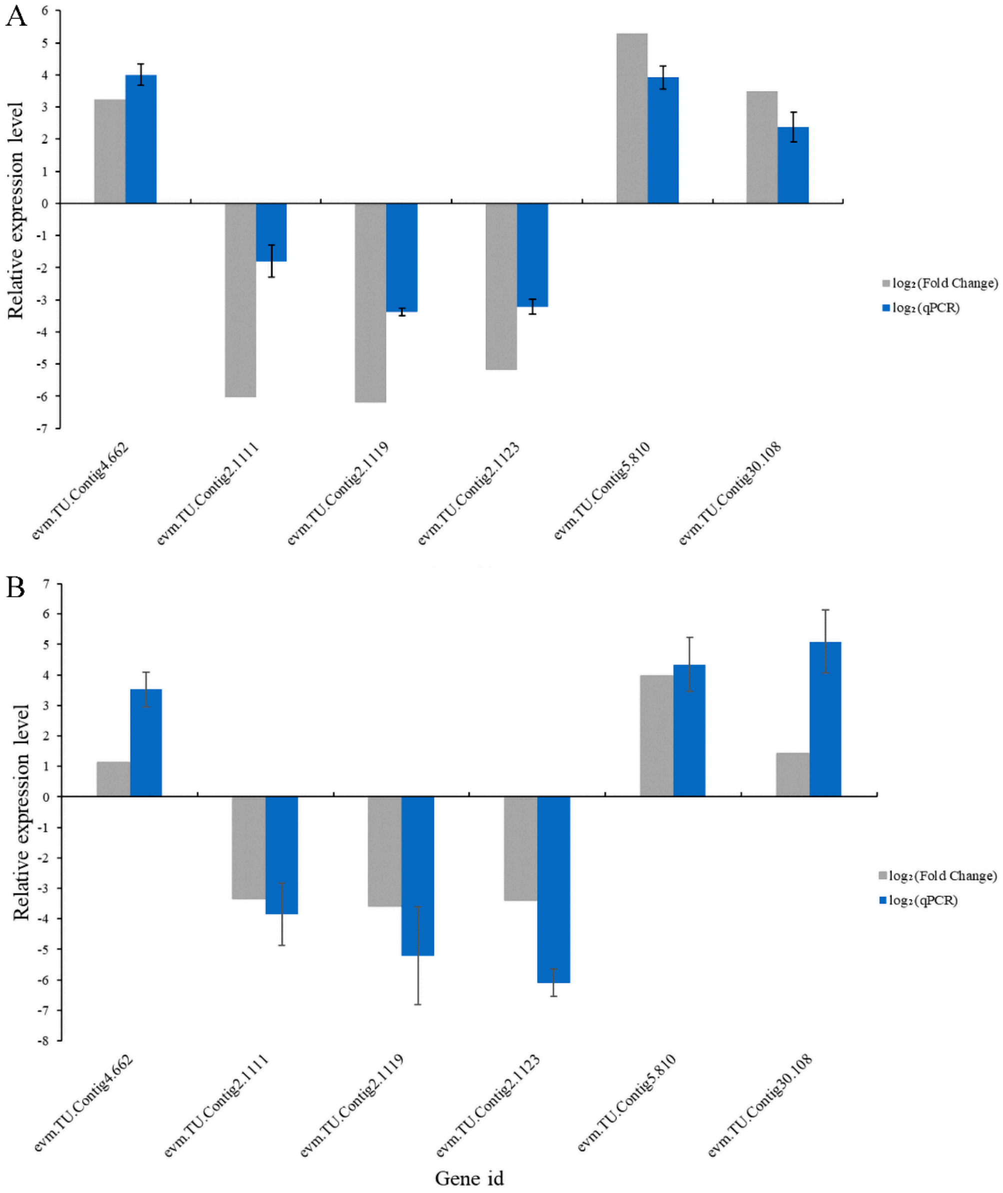

Figure S5. Relative expression level of 6 DEGs in (A) BF_R4d vs R4d and (B) BF_R7d vs R7d using reference gene β-actin for normalization by qRT-PCR included evm.TU.Contig4.662 (endo-1,4-beta-xylanase 1), evm.TU.Contig2.1111 (highly reducing polyketide synthase azaB), evm.TU.Contig2.1119 (acetohydroxy-acid synthase catalytic subunit AHAS), evm.TU.Contig2.1123 (putative sterigmatocystin biosynthesis peroxidase stcC), evm.TU.Contig5.810 (multidrug resistance protein CDR2), and evm.TU.Contig30.108 (pectate lyase). The X-axis indicates the name of the DEGs, and the Y-axis indicates the relative expression of the DEGs.
